# Collaborative, digital and sustainable skills through multimodal simulations: evidence-based, interprofessional training in primary health care – the REALISE study

**DOI:** 10.1186/s41077-026-00468-1

**Published:** 2026-07-22

**Authors:** Christine Kern, Melanie Roth, Marlene Brunner, Eva Maria Bamberger, Joachim Schulze, Gertie Janneke Oostingh, Daniela Huber

**Affiliations:** Department of Health Sciences, University of Applied Science Salzburg, Urstein Sued 1, 5412 Puch/Salzburg, Austria

**Keywords:** Interprofessional collaboration, Interprofessional education, Primary health care, Health Personnel, Social Work, Simulation Training

## Abstract

**Background:**

Interprofessional collaboration is crucial for enhancing healthcare delivery, particularly in primary care settings, where effective teamwork between healthcare and social professionals (HCSPs) is essential. Despite its importance, there is limited research on the impact of structured interprofessional training programs on interprofessionality in simulated environments, particularly concerning primary care contexts.

**Methods:**

The REALISE study constitutes of a prospective feasibility study examining the impact on interprofessionality of multimodal, interprofessional learning paths on HCSPs as well as students within a simulated primary healthcare (PHC) setting. The three-week learning paths were specially developed, proprietary, case-oriented, and consisted of simulations (acting patients, high-fidelity manikins, virtual reality) and didactic on- and offline elements. The study employed a mixed-methods approach, incorporating three conducted interprofessional intervention groups to evaluate outcomes across both quantitative (two standardized assessments: Interprofessional Collaborative Competency Attainment Survey (ICCAS), Interprofessional Socialization and Valuing Scale 9a&b (ISVS)) and qualitative (proprietary open-ended questions) domains. Data was collected before (t0), directly after (t1) and two months after t1 (t2) to evaluate the impact on interprofessional collaboration.

**Results:**

Regarding the quantitative outcomes, no significant change was observed in participants’ ISVS scores between pre- and post-intervention assessments, neither in the entire sample (t0 = 5.41 ± 1.20, t1 = 5.85 ± 1.18; *p* = 0.108), nor in the student or HCSP group. Also, no time*group effects became evident. This suggests stable self-perceived values and attitudes toward interprofessional collaboration. In contrast, ICCAS scores increased significantly in total and across all dimensions (e.g., total score t₀ = 3.00 ± 0.77, t₁ = 3.99 ± 0.53; *p* < 0.001, *d* = 0.91). The strongest effects for the entire sample were observed in the dimensions “Team Functioning” (0.973) and “Collaboration” (0.922) and significant time*group (students vs. HCSPs) effects were found in 3 of the 6 dimensions (“Collaboration”, “Roles and Responsibilities” and “Team Functioning”), indicating substantial self-reported improvements in interprofessional competencies following the training and differences in amplitude of improvements. The results of the qualitative thematic analysis showed clear differences and overlaps between groups and time points. At t0, the students described communication difficulties, unclear responsibilities and role confusion as central challenges. In contrast, HCSPs focused on structural problems such as time pressure and limited coordination. At t1, the students reported increased role clarity and improved team communication, while HCSPs noted greater awareness of their own role and greater flexibility in interdisciplinary cooperation. As part of the follow-up (t2) the HCSPs reported a further deepening of interprofessional cooperation in their daily practice.

**Conclusions:**

While participants’ self-perceived attitudes remained stable, significant improvements in interprofessional competencies such as team functioning and collaboration, showed that the training effectively supported collaborative practice. The intervention was feasible to implement and was positively evaluated across all professional groups.

**Supplementary Information:**

The online version contains supplementary material available at https://doi.org/10.1186/s41077-026-00468-1.

## Highlights

What are the main findings?

- Simulation-based interprofessional training within the REALISE study resulted in* significant* improvements in core collaborative competencies, particularly in the domains of“Team Functioning” and “Collaboration” as measured by the ICCAS instrument.
- While participants’ self-perceived attitudes toward interprofessional collaboration, as assessed by ISVS, remained stable, the substantial gains in applied competencies and the overall acceptance of the training underscore its educational relevance and perceived impact.
- Students reported gaining deeper insight into other professional roles and greater role clarity, while HCSPs described perceived improvements in collaborative practice, increased sensitivity to communication processes, and greater openness toward the competencies of other professions, as reflected in qualitative responses and ICCAS domain-specific gains

What are the implications for (continued) education?

- The findings affirm the feasibility of implementing high-quality interprofessional simulation training, even among newly formed and professionally diverse participant groups who do not yet typically work together in real-life settings, in Austria.
- Sustainable integration into healthcare education and practice requires highly qualified facilitators, institutional support, and ongoing curricular refinement to ensure long-term efficacy across diverse clinical environments.

**Supplementary Information:**

The online version contains supplementary material available at https://doi.org/10.1186/s41077-026-00468-1.

## Introduction

Given the increasing complexity of healthcare, effective care delivery now depends on coordinated team efforts rather than individual performance [1]. Interprofessional learning (IPL) and interprofessional education (IPE) aim to strengthen collaboration among healthcare and social professionals (HCSPs), thereby improving care quality, efficiency, and coordination [2–5]. These approaches foster mutual trust, respect, and recognition of diverse competencies, while challenging professional stereotypes and hierarchies [6–8].

Evidence links IPL and IPE to improved organizational performance, workforce well-being, and patient-centred outcomes, including improved interprofessional coordination, increased patient satisfaction, cost efficiency, and enhanced team dynamics [2, 9, 10]. The relevance of interprofessional approaches is further underscored by current demographic trends, the rising prevalence of chronic diseases, and persistent shortages in the healthcare workforce [3]. Even though educational and clinical implementations are becoming established [11, 12], constraints, such as insufficient time and training, unclear professional roles, concerns about professional identity, and ineffective interprofessional communication hinder successful collaborative practice [13]. While early evidence supports the efficacy of IPE and IPL – particularly in improving attitudes, collaborative behaviours, and team-based clinical practices – interprofessional models are not yet systematically embedded in HCSPs education or clinical routine, indicating a need for rigorous evaluation, theoretical refinement, and curricular integration [11, 14].

Within this context, simulation-based training serves as an ideal learning element of IPL, facilitating the integration of theoretical knowledge and practical application in a safe environment. This structured, experiential approach bridges the gap between academic instruction and clinical practice [15–17]. By replicating realistic clinical scenarios in a controlled, risk-free setting, simulation enables learners to apply knowledge to practical decision-making processes without compromising patient safety and allows for repetition to optimize performance [15–17]. Due to its adaptable and immersive nature, healthcare simulation is increasingly recognized as a powerful platform for interprofessional education, enabling tailored, role-inclusive experiences that foster collaborative care through practice and reflection [1].

Studies have shown that IPE simulations can improve students' self-confidence, interprofessional communication skills, and readiness for collaborative practice [18, 19]. Furthermore, these simulations provide a safe environment to practice teamwork and develop a better understanding of other professionals' roles [20, 21]. In addition, in its 2018 guidelines [22], the World Health Organization explicitly recommends the integration of simulation-based education into HCSPs training. Institutions should adopt simulation modalities that are appropriate to their contextual resources to enhance the acquisition of clinical competencies and patient safety practices.

IPE simulations involving students from various health disciplines significantly enhances cultural competence, role clarity, communication, and team collaboration [23], highlighting the pedagogical value of simulation. Effective implementation, however, requires clearly defined learning objectives, realistic scenarios, and structured feedback to optimize educational outcomes [21]. Integrating simulations into healthcare curricula may enhance collaborative practice readiness and contribute to improved patient safety [24]. Beyond that, HCSPs in practice require ongoing educational opportunities to further refine their interprofessional competencies [25]. Austrian HCSPs in primary healthcare (PHC) highlight the need for targeted Interprofessional collaboration (IPC) training to address complex clinical demands [26].

From a PHC perspective, additional systemic challenges become evident. Globally, PHC systems are under growing pressure to respond to the changing health needs of populations, driven by ongoing demographic and epidemiological transitions. Persistent issues include workforce shortages, insufficient training, and limited adaptability to the rising burden of non-communicable diseases [27, 28]. Additional challenges include fragmented care, staff burnout and retention [29], and the disruptive effects of digital transformation [30]. Quality assurance and patient safety concerns further hinder PHC performance [31]. Nevertheless, strengthening PHC remains essential for managing chronic conditions effectively [32].

To address these challenges, structural transformation and context-specific, innovative care models are essential to enhance PHC systems’ efficiency and resilience. [33, 34]. Through high-fidelity, contextually relevant simulations, participants not only enhance their clinical proficiency but also acquire vital competencies in communication and stress resilience. This contributes to improved quality of care, heightened patient safety, and the reinforcement of PHC as a foundational pillar of the health system. By addressing gaps in continuing professional development, such training also alleviates pressure on secondary and tertiary care levels [35].

This underscores the urgent need to advance simulation-based training by aligning it with the evolving demands of PHC, particularly through digital formats in resource-limited settings. Greater emphasis should also be placed on interprofessional, primary care-oriented education for students.

Therefore, the purpose of the REALISE study (“Collaborative, Digital and Sustainable Skills through Multimodal Simulation: Evidence-Based, Interprofessional Training in Primary Healthcare”) is to explore how the integration of collaborative, digital, and sustainability elements in multimodal learning paths enhances the development of interprofessional competencies within primary health- and social care education.

The study objectives are the following:

a. to evaluate the impact of multimodal, simulation-based interprofessional training on participants’ interprofessional competencies, as assessed through validated quantitative instruments (Interprofessional Collaborative Competency Attainment Survey (ICCAS), Interprofessional Socialization and Valuing Scale (ISVS)).

b. and to explore participants’ perceptions of interprofessional collaboration, role clarity, and related behavioural changes before and after the intervention, using qualitative open-ended responses.

## Materials and methods

### Trial design

The REALISE study (https://doi.org/10.17605/OSF.IO/9USC5) was a prospective feasibility study investigating the effects of learning paths (multimodal, interprofessional, setting: PHC) on interprofessionality in two cohorts: HCSPs and students. Three parallel mixed-cohort intervention groups were conducted. The study protocol was approved by the Institutional Ethics Committee of the University of Applied Science Salzburg, Austria (reference number 2024-GW-001). The design and detailed account of the methodology was previously published [36].

### Participants recruitment

The REALISE study included adults (18–68 years) who were either employed or final-year students in one of 16 predefined health and social care professions: biomedical sciences, dietetics, general medicine, medical assistance, medical training therapy, midwifery, nursing, occupational therapy, orthoptics, paediatrics, pharmacology, physiotherapy, psychology, psychotherapy, radiology technology, speech and language therapy, and social work. Professionals required at least two years of PHC or related experience and regular interprofessional patient contact.

Recruitment of participants took place from May until September 2024 via various channels. 39 stakeholders and professional associations were contacted directly and invited to disseminate information on study participation. In addition, information on study participation was sent by email to the 53 primary care units in Austria (as of May 2024), the 124 rehabilitation facilities in Austria (as of June 2024) and the 115 GP practices with existing email addresses in the province of Salzburg and the neighbouring regions (Upper Austria, Carinthia, Tyrol, East Tyrol and Bavaria). Furthermore, flyers were distributed at events and conferences and digital channels such as the Austrian primary care platform, LinkedIn, Facebook and Instagram were used to draw attention to the study. At the same time, the educational institutions of the 16 professional groups in Salzburg and the neighbouring Austrian provinces were asked to invite their students to take part in the study.

### Participant allocation

**Fig. 1:**
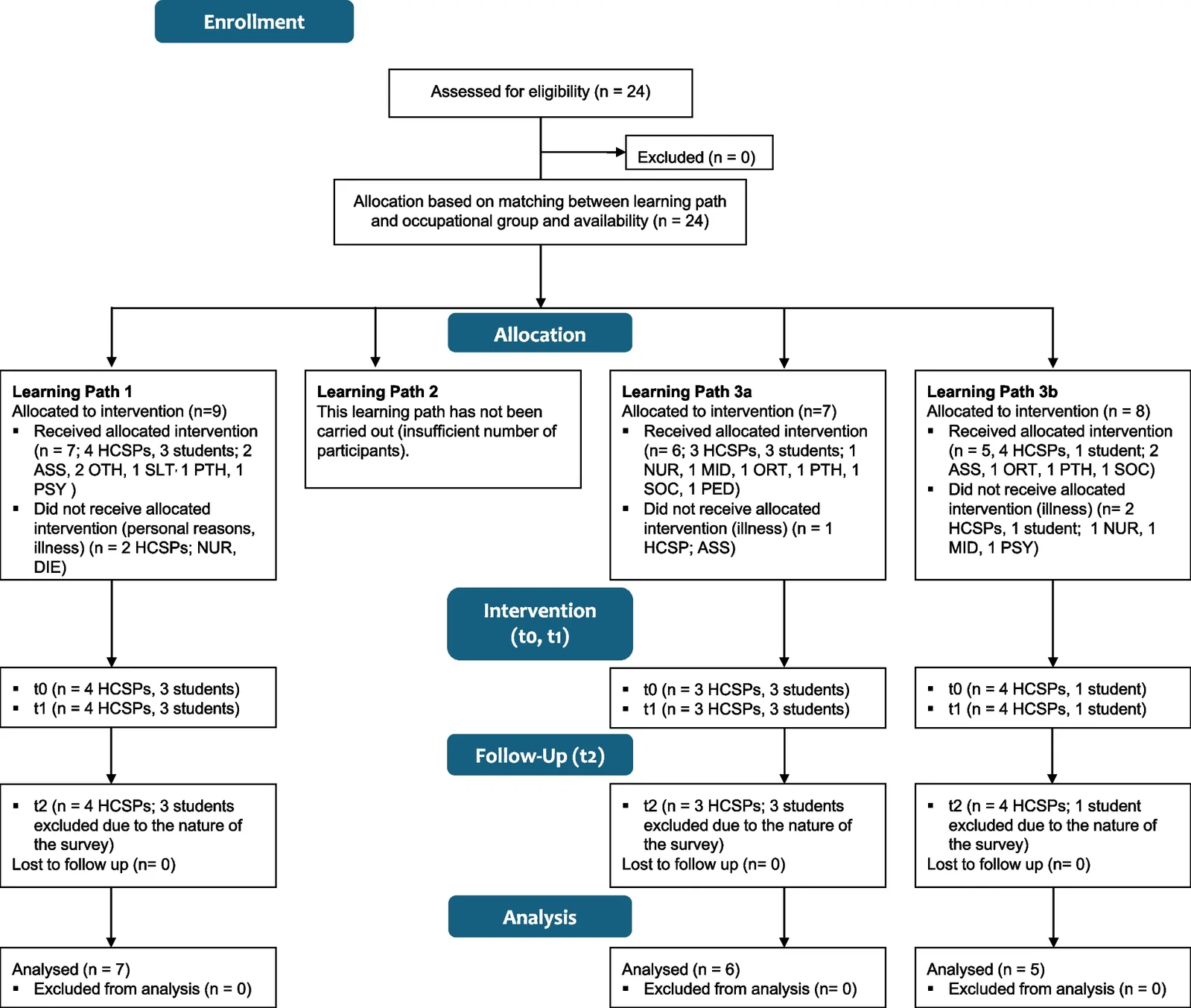
Participant flow through learning paths and inclusion in analysis (per CONSORT statement 2025 [37]). Abbreviations: dietologist (DIE), medical assistant (ASS), midwife (MID), nurse (NUR), occupational therapist (OTH), orthoptist (ORT), paediatrician (PED), physiotherapist (PTH), psychotherapist/psychologist (PSY), speech and language therapist (SLT), social worker (SOC), healthcare and social professionals (HCSPs)

A total of 24 participants, meeting all inclusion and exclusion criteria, volunteered to take part in the study and were subsequently allocated to one of three distinct learning paths. Details to participant flow are shown in (Fig. 1).

**Fig. 2:**
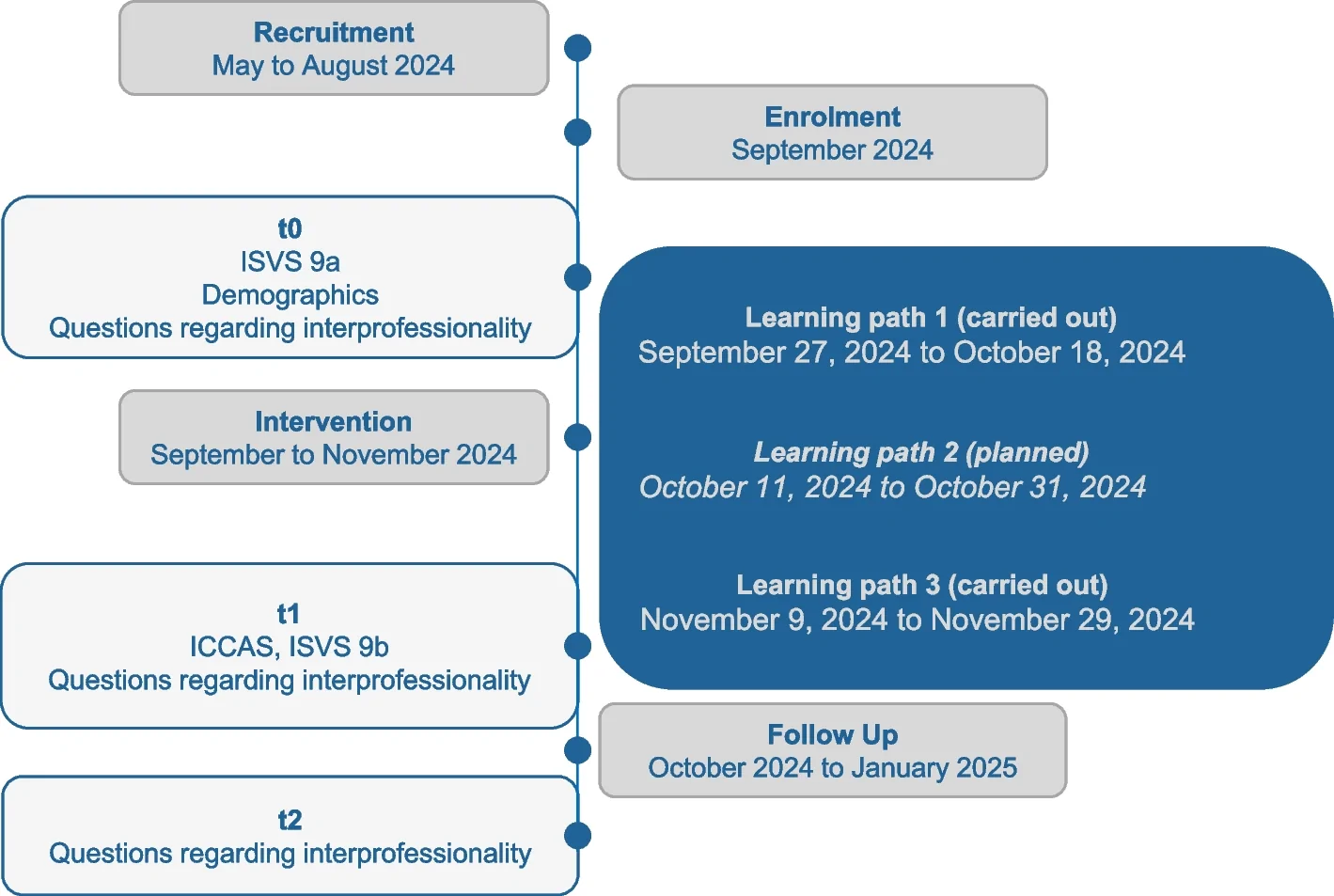
Study schedule regarding relevant outcomes for this manuscript

Nine individuals (six HCSPs, three students) were allocated to learning path 1, two did not receive the intervention. The implementation of learning path 2 was unsuccessful due to an insufficient number of participants. Given the excessive number of participants enrolled in learning path 3, it was subsequently divided into two distinct conducts, designated as 3a and 3b, respectively. Seven individuals (four HCSPs, three students) were enrolled in learning path 3a, one did not receive the intervention. Eight individuals (six HCSPs, two students) were enrolled in learning path 3b, three did not receive the intervention. The analysis encompassed all included HCSPs (*n* = 11) and students (*n* = 7) and at t0 and t1, and all HCSPs at t2. No losses were identified for in follow-up (Fig. 1). The intervention phase took place from September to November 2024, followed by a follow-up two months after the intervention (Fig. 2).

### Interventions

Participants engaged in four in-person eight-hour days over a three-week period. In addition, blended learning was implemented, whereby participants, based on their individual professional needs and interests, completed a minimum of four hours per week through E-Learning (including presentations, videos, and assignments) and a digital communication tool (Medikit tool, hotelkit GmbH, Salzburg/Austria, https://medikit.net/de/). The in-person days, conducted at the Salzburg University of Applied Sciences in Puch/Urstein (Austria, 47.727128° N, 13.085423° E), were designed for interprofessional groups of 7–10 individuals, consisting of students and HCSPs. The study incorporated six case scenarios focused on PHC, with two cases per learning path, integrating simulations with standardized patients, high-fidelity manikins and immersive virtual reality environments. The case scenarios encompassed eleven key areas of PHC [38], including health promotion, outpatient care, rehabilitation, long-term care, and palliative care. Patient examples, focusing on non-communicable diseases and the impact of climate change, included cases about a geriatric couple with internal problems and a young mother post-stroke (learning path 1), an older woman with acute coronary syndrome and a young male athlete with Long Covid (learning path 2) as well as a pregnant woman with pre-eclampsia and a middle-aged man with diabetes (learning path 3). A detailed description of the simulation can be found in Supplemental Table S1 in accordance with the reporting guideline by Cheng et al. [39]. To promote psychological safety during simulation-based learning, several structured strategies were employed. All simulations were facilitated by trained instructors with formal experience in IPE and simulation-based teaching. Each simulation day began with a standardized pre-briefing, during which learning objectives, roles, and expectations were clarified, and explicit ground rules were established, including confidentiality, mutual respect, and the normalization of errors as learning opportunities. Debriefings followed a structured, facilitator-led format focusing on reflective dialogue, shared sense-making, and non-judgmental feedback, thereby supporting open communication and interprofessional trust.

Furthermore, the simulations were complemented by in-person didactic elements.

### Outcomes

Assessments were carried out at t0 (pre-intervention) and t1 (post- intervention) at the Salzburg University of Applied Sciences, with a follow-up (t2 = t1 + 60 days) via online survey. An overview of all outcomes collected within the REALISE study can be found in the previously published study protocol [36]. Only outcomes relevant to this manuscript, will be elaborated on further:

Primary outcomes included the following two validated instruments for both cohorts:

1. ISVS 9a (at t0)/9b (at t1) [40], in the German version [41]: The ISVS 9a (t0) and 9b (t1) were analysed in accordance with the described protocol [42]. The ISVS utilises a 7-point Likert scale. Higher values indicate a positive change in interprofessional socialization after interprofessional education.
2. The revised ICCAS (at t1) [43, 44], in a German translated version [45]: The revised ICCAS utilises a 5-point Likert scale to evaluate the perceived attainment of interprofessional collaborative competencies. Higher scores indicating greater perceived competence in its dimensions. The instrument incorporates a retrospective pretest design, enabling participants to rate their competencies both before and after the intervention at a single measurement point after the intervention. Data was analysed according to Archibald et al. [46].

Secondary outcomes for both cohorts were a set of 3 open-ended, proprietary questions regarding interprofessionality at t0 and t1. At t2, only HCSPs responded to an additional set of 3 open-ended, proprietary questions to assess longer-term developments in practice (Table 1). This approach allowed for a nuanced interpretation of changes in thinking and practice without quantification [47].

**Table 1:** Interprofessionality-Related Questions Administered at t0, t1, and t2

Timepoint	Cohort	Question
t0	HCSPs, students	What challenges do you face when integrating interprofessional aspects into your daily work?
t0	HCSPs, students	How could your tasks and roles change as a result of improved interprofessional collaboration?
t0	HCSPs, students	What support do you expect from the training modules to promote interprofessional collaboration?
t1	HCSPs, students	What challenges do you now face when integrating interprofessional aspects into your daily work?
t1	HCSPs, students	How have your tasks and roles changed as a result of improved interprofessional collaboration?
t1	HCSPs, students	To what extent have the training modules helped you to promote interprofessional collaboration?
t2	HCSPs	Has your perception of interprofessional collaboration changed in your practical work? If so, how?
t2	HCSPs	Were you able to apply content related to interprofessional collaboration?
t2	HCSPs	Was there a change in your tasks and roles?

*Abbreviations*: *HCSPs* healthcare and social professionals

### Randomisation, sample size and blinding

The sample size was designed in accordance with the following recommendations to ensure active participation from all team members during the simulation: Nehring et al. [48] observed that group sizes exceeding 8 to 10 students hinder participants to effectively interact with both the patient and the simulation equipment, as well as to adequately observe these elements [1]. Au et al. [49] identified an optimal group size of 5 to 6 participants to maximize the efficacy of high-fidelity. Consequently, the sample size of 7–10 participants per training module was consistent with these recommendations; as not everyone was actively involved in the simulations at the same time; instead, some participants served as observers and provided feedback.

An allocation scheme was developed for targeted recruitment, based on the content-related alignment of the professional groups with the case scenarios within the respective learning paths (Supplemental Table S2). One position was allocated to students and one to HCSPs for each learning path. In cases of sufficient participation interest, stratification was performed according to gender, age and professional experience. Owing to the nature of the intervention, blinding could not be implemented. The sample constituted a convenience sample based on participant availability during the study period. No formal a priori sample size calculation or effect size estimation was conducted, given the exploratory, feasibility-oriented nature of the study.

### Statistical and qualitative data analysis

For quantitative data, intragroup comparisons were performed using the Fisher's exact test, paired t-test, and the Wilcoxon signed-rank test. Comparisons between groups were conducted using Fisher's exact test and an aligned rank transform analysis of variance (ART ANOVA). The significance threshold was set at *p* < 0.05. All statistical analyses were carried out using R software (R Foundation, Vienna, Austria; https://www.r-project.org/), in accordance with per-protocol principles.

For qualitative data, the analysis was based on a qualitative content analysis according to Mayring [50]. The data were analysed deductively, using predefined questions based on theoretical concepts of interprofessional practice [50–53]. The methodological approach followed the recommendations of Flick [47], with central quality criteria of qualitative research, particularly transparency, traceability, and a rule-governed, theory-based approach, being considered.

## Results

### Participant characteristics

Eighteen individuals (15 female, 3 male) completed the study. Age distributions differed between groups: students were predominantly younger, with 57% being under 25 years of age, whereas HCSPs were mainly between 36 and 55 years, with the largest proportions in the 36–45 and 46–55 age groups (36% each). The mean professional experience of the HCSPs was 11.45 years (SD 8.19), with a median of 12 years. Two (18%) of the HCSPs were working in leadership roles and nine (82%) as team members. Students were enrolled in the senior year of their degree programs at the time of data collection and, as expected, reported neither professional work experience nor leadership responsibilities. With exception of the highest professional education, students and HCSPs do not differ in the recorded demographics. Detailed demographic information is presented in Table 2.

**Table 2:** Participants’ characteristics

		**Total sample (*n* = 18)**	**Students (*n* = 7)**	**HCSPs (*n* = 11)**	***p****-values $*
		absolute (relative)	absolute (relative)	absolute (relative)	
**Gender**	female	15 (83%)	5 (71%)	10 (91%)	0.528
	male	3 (17%)	2 (29%)	1 (9%)	
	non-binary	0 (0%)	0 (0%)	0 (0%)	
	prefer not to say	0 (0%)	0 (0%)	0 (0%)	
**Age**	< 25	4 (22%)	4 (57%)	0 (0%)	0.069
	26–35	3 (17%)	1 (14%)	2 (18%)	
	36–45	5 (28%)	1 (14%)	4 (36%)	
	46–55	5 (28%)	1 (14%)	4 (36%)	
	56–65	1 (6%)	0 (0%)	1 (9%)	
	> 66	0 (0%)	0 (0%)	0 (0%)	
**Highest Educational Qualification**	without high school diploma/A-levels*	4 (22%)	0 (0%)	4 (36%)	**0.007**
	with high school diploma/A-levels*	5 (28%)	5 (71%)	0 (0%)	
	Bachelor	1 (6%)	0 (0%)	1 (9%)	
	Master	7 (39%)	2 (29%)	5 (45%)	
	PhD/Doctorate	1 (6%)	0 (0%)	1 (9%)	
**Position**	Leadership role	2 (11%)	0 (0%)	2 (18%)	0.497
	Team member	16 (89%)	7 (100%)	9 (82%)	
**Work location**	Eastern Austria	1 (6%)	0 (0%)	1 (9%)	1
	Western Austria	17 (94%)	7 (100%)	10 (91%)	
**Profession**	Medical Assistant	4 (22%)	0 (0%)	3 (36%)	0.072
	Midwife	1 (6%)	0 (0%)	1 (9%)	
	Nurse	1 (6%)	1 (14%)	0 (0%)	
	Occupational Therapist	2 (11%)	2 (29%)	0 (0%)	
	Orthoptist	2 (11%)	0 (0%)	2 (18%)	
	Paediatrician	1 (6%)	0 (0%)	1 (9%)	
	Physiotherapist	3 (17%)	1 (14%)	2 (18%)	
	Psychotherapist	1 (6%)	1 (14%)	0 (0%)	
	Speech and Language Therapist	1 (6%)	0 (0%)	1 (9%)	
	Social Worker	2 (11%)	2 (29%)	0 (0%)	

*Abbreviations*: * Matura in Austria, *HCSPs* healthcare and social professionals, $ Fisher’s Exact test; in bold: statistically significant values

### Primary (quantitative) outcomes

#### ISVS

The mean ISVS score at t0 in the entire sample was already relatively high, with 5.41 ± 1.20 point on the 7-point Likert scale. The increase to t1 (t1 = 5.85 ± 1.18) did not reach statistical significance (*p* = 0.108, paired t-test, Cohens D = 0.40, CI = [−1.00; 0.11]). HCSPs improved from 5.42 ± 1.18 to 5.75 ± 1.25 (*p* = 0.364, paired t-test, Cohens D = 0.29, CI = [−1.08; 0.44]) and students from 5.38 ± 1.23 to 6.02 ± 1.06 (*p* = 0.193, paired t-test, Cohens D = 0.55, CI = [−1.70; 0.43]).The increases in the two cohorts also did not show a time*group effect (*p* = 0.977, ART ANOVA, Cohens D = 0.29). Group-specific differences in change over time were examined using a non-parametric mixed-design ANOVA, based on the Aligned Rank Transform (ART), with time as a within-subject factor and professional background as a between-subject factor. No significant time*group interaction was detected (*p* = 0.977), suggesting that the observed increases did not differ meaningfully between cohorts. This suggests that while a positive trend was observed, the change in self-perceived interprofessional socialization was not statistically significant in this sample.

#### ICCAS

The mean total ICCAS score started in a middle range on a 5-point Likert scale (t0 = 3.00 ± 0.77) and improved significantly to t1 (3.99 ± 0.53) (*p* < 0.001, Wilcoxon signed-rank test, CI = [1.00, 1.50]) in the entire sample. Specifically, HCSPs improved from 3.16 ± 0.68 to 4.04 ± 0.46 (*p* < 0.001, Wilcoxon signed-rank test, CI = [1.00, 1.00]) and students from 2.74 ± 0.91 to 3.92 ± 0.63 (*p* < 0.001, Wilcoxon signed-rank test, CI = [1.49, 1.50]). To examine whether changes over time differed between professional backgrounds, a non-parametric mixed-design ART was conducted, modelling time as a within-subject factor, professional background (HCSPs vs. students) as a between-subject factor, and including a random intercept for subjects to account for repeated measurements. For the total score no significant time*group interaction was observed (*p* = 0.077). All increases in the dimensions were statistically significant (*p* < 0.001, Wilcoxon signed-rank test). The strongest effects for the entire sample were observed in the dimensions “Team Functioning” (Cohens D = 0.973) and “Collaboration” (Cohens D = 0.922) and significant time*group effects (ART ANOVA) were observed in the dimensions “Collaboration”, “Roles and Responsibilities” and “Team Functioning” with students starting consistently in lower mean values in these dimensions than HCSPs, see Table 3. This indicates that participants perceived a meaningful improvement in interprofessional competencies following the intervention.

**Table 3:** ICCAS score development by group and dimension (Before–After)

	**Total Sample mean (SD) §**		**Cohens D [CI]**	**Student mean (SD) §**		**Cohens D [CI]**	**HCSP mean (SD) §**		**Cohens D [CI]**	Time*group effect &
	**before**	**after**		**before**	**after**		**before**	**after**		*p*-value
Total score	3.00 (0.77)	3.99 (0.53)	0.909 [1.00,1.50]	2.74 (0.91)	3.92 (0.63)	0.893 [1.49,1.50]	3.16 (0.68)	4.04 (0.46)	0.926 [1.00,1.00]	0.077
Dimension Communication	3.09 (0.70)	3.94 (0.36)	0.909 [1.00,1.50]	3.00 (0.72)	3.89 (0.46)	0.893 [1.00,1.50]	3.15 (0.69)	3.98 (0.29)	0.926 [1.00,1.50]	0.264
Dimension Collaboration	2.83 (0.49)	4.00 (0.52)	0.922 [1.00,1.50]	2.38 (0.61)	3.95 (0.77)	0.935 [1.00,2.00]	3.12 (0.42)	4.03 (0.35)	0.917 [1.00,1.50]	**0.017**
Dimension Roles and Responsibilities	3.13 (0.72)	4.24 (0.40)	0.901 [1.50,1.50]	3.75 (0.95)	4.05 (0.37)	0.893 [n/a]	3.36 (0.57)	4.25 (0.42)	0.934 [1.00,1.50]	**0.014**
Dimension Collaborative Patient/Family-Cantered Approach	2.76 (0.66)	3.87 (0.35)	0.896 [1.00,1.50]	2.38 (0.95)	3.67 (0.41)	0.904 [1.00,2.00]	3.00 (0.47)	4.00 (0.31)	0.908 [1.50,1.50]	0.181
Dimension Conflict Dimension Management/Resolution	3.39 (0.59)	4.00 (0.34)	0.903 [n/a]	3.57 (0.64)	4.05 (0.47)	0.900 [n/a]	3.27 (0.56)	3.97 (0.26)	0.920 [n/a]	0.210
Dimension Team Functioning	2.53 (0.20)	3.81 (0.35)	0.973 [1.00,1.50]	1.93 (0.10)	3.57 (0.61)	0.999 [1.00,2.50]	2.91 (0.26)	3.95 (0.19)	0.962 [n/a]	0.049

*Abbreviations*: *CI* Confidence Interval, *n/a* not applicable, *SD* Standard Deviation

*§  p*-value < 0.001 Wilcoxon signed-rank test, & ART ANOVA; in bold: statistically significant values

### Secondary (qualitative) outcomes

The summary of responses was based on predefined categories derived from the research questions in a structured manner, while remaining open to differentiating scenario perspectives. The before-and-after design aligns with the logic of a qualitative longitudinal study: rather than aiming for numerical comparability, the aim was to present participants shifts in meaning and professional behaviour.

Findings revealed before the training (t0) clear thematic differences and overlaps between groups and time points. Students (S) mainly described communication difficulties, unclear responsibilities, and role confusion as key challenges: *"little idea of what the other profession actually does" (t0,S23)*. HCSPs (H), in contrast, focused on structural issues such as time constraints and limited coordination: “*time resources are often very limited” (t0,H7).* After the training (t1), students reported increased role clarity and improved team communication:*”interlocking competencies within the team for the benefit of patients” (t0,S23),* while HCSPs noted a stronger awareness of their own roles and greater flexibility in collaborating across disciplines: *“I pay more attention to recognizing opportunities for interprofessional collaboration” (t1,H24).*

Regarding expectations and impact of the training, students emphasized gaining deeper insight into other professions and valued the communication tools provided: *“communication training helped to communicate more clearly” (t1,S5).* HCSPs reported that simulations and structured reflection helped them enhance their collaborative practice. Some participants described concrete behavioural changes, such as more open communication and clearer role awareness: *“I will more often seek direct conversations or phone calls and approach other professions more openly” (t1,H22)*, while others cited limited opportunities to apply what they had learned: *“At present, there is no opportunity to apply this in my professional practice” (t1,H17).*

As part of the follow-up phase (t2), HCSPs reported further consolidation of interprofessional collaboration in their daily practice: *“working more strongly as a team and moving in the same direction” (t2,H4)*. Several participants described increased sensitivity to communication processes, greater openness towards the competencies of other professions, and the practical implementation of structured handover tools: *“structured hand over, team leadership, and clear role distribution” (t2,H1).* Although some structural limitations persisted, the overall perception of IPC’s relevance and applicability had clearly strengthened.

## Discussion

The varied professional composition of participants demonstrates that multimodal simulation-based training is feasible for diverse interprofessional teams in primary health and social care. This diversity aligns well with real-world team structures and strengthens the ecological validity of the intervention. Participants’ educational backgrounds ranged from no secondary school certificate (Matura) to doctoral degrees. This variability requires an instructional design that accommodates different levels of prior knowledge and academic preparation. Consequently, the learning paths must be pedagogically flexible and inclusive [54]. In parallel, the marked predominance of female participants (83%) reflects prevailing gender distributions within health and social care professions in Austria [55]. While this enhances the representativeness of the sample, it simultaneously prompts reflection on the gendered dimensions of interprofessional training environments, including the potential need for tailored approaches to better engage underrepresented demographic groups [56, 57].

Furthermore, the disparity in professional experience – ranging from a median of 0 years among trainees to 12 years among practicing health professionals (*p* = 0.001) – underscores a fundamental divergence in educational needs, as learning outcomes in IPE are known to vary depending on learner characteristics and context [58]. While early-stage learners may derive maximal benefit from foundational exposure to IPC, experienced practitioners are likely to require more advanced, context-specific content to perceive the training as both relevant and applicable to their clinical roles [59]. This diversity in background and experience likely shaped the learning experience: it enriched discussions, role-play exercises, and peer learning by bringing multiple perspectives, but also posed challenges in pacing and content complexity [60]. Some participants needed more foundational guidance, while others sought advanced, practice-oriented scenarios. Future programs could address these differences through differentiated learning pathways, preparatory materials, or small-group sessions tailored to experience levels, aligning with recommendations for learner-centred and level-appropriate simulation design [61].

Moreover, the inclusion of both students and experienced HCSPs in mixed cohorts may have shaped behavioural dynamics and professional identity formation – particularly among students [62]. While the presence of seasoned practitioners plausibly facilitated peer learning, it may also have influenced students’ perceptions of interprofessional roles and collaboration, potentially amplifying or attenuating training effects [62]. This intentional mix could be seen as a notable strength, as this heterogeneity fostered mutual learning and exchange across experience levels [62]. These interpretations are hypothesis-generating and warrant explicit examination in future studies (e.g., comparative cohort designs) to disentangle effects on learning outcomes, identity development, and attribution of behavioural change to programme components. The positive outcomes observed despite these contextual challenges indicate feasibility within a simulated PHC setting and reflects the intended dual applicability of the modules across undergraduate education and continuing professional development. In this context, the findings are particularly relevant for forthcoming programme reforms at the Health Sciences Programme at the University of Applied Sciences Salzburg, which will establish a clearer structural delineation between initial professional education and continuing education. Future cohorts will therefore consist either of learners in initial training or practicing HCSPs. This restructuring is expected to reduce heterogeneity in professional experience and support more coherent curriculum development and pedagogical alignment.

Although the simulation component represented only a relatively small proportion of the overall multimodal programme, it served as a central experiential anchor for IPL [63]. The simulations enabled participants to actively engage in role enactment, structured reflection, and collaborative problem-solving within realistic PHC scenarios. While the quantitative contribution of the simulation to overall competency gains cannot be fully disentangled from other programme elements, the qualitative data indicate that simulation provided a crucial space for practicing communication, clarifying roles, and testing IPC strategies in a safe environment [60], which likely facilitated the transfer of learning to real-world PHC contexts. Accordingly, particular attention was devoted to the qualitative feedback of participants and instructors to assess, as far as possible, the perceived contribution of specific programme components to reflections and behavioural changes.

While the study encompassed a broad spectrum of HCSPs, some professions, particularly those primarily associated with rehabilitation clinics, may not universally be considered part of primary healthcare in international contexts. Nonetheless, their inclusion reflects the real-world complexity and interprofessional nature of healthcare delivery in Austria, and highlights the value of preparing teams for collaborative practice across settings that bridge primary, community, hospital and tertiary care [64]. Moreover, differences in how primary healthcare is structured internationally may limit the transferability of findings [65], particularly regarding team composition, scope of practice, and interprofessional collaboration dynamic.

The significance of the ICAAS results can be attributed to its specific focus on actual collaboration and concrete behavioural changes within interprofessional teams. Methodologically, the ICCAS retrospective pretest may facilitate more accurate recognition of overestimated IPC competencies and attenuate ceiling effects relative to the prospective ISVS. Notably, a recent validation study demonstrated that retrospective pretest measures in the ICCAS exhibit robust reliability and validity, with no significant differences compared to traditional pretest–posttest designs, thereby mitigating concerns related to recall or social desirability biases [66].

In contrast, ISVS primarily measures professionals' perceptions and attitudes towards collaboration, which were already high at baseline. In addition, at baseline (t0), participants may not yet be fully aware of their own unacknowledged gaps in understanding IPC [67].

For instance, ISVS item 9a states, “I have gained an enhanced awareness of roles of other professionals on a team” – a level of insight that some participants may only truly develop once they are required to articulate and reflect on another profession’s role during the first simulation day.

The qualitative analysis indicates that training and continuing education can contribute to enhanced interprofessional communication, clearer role delineation, and more efficient work processes. However, challenges such as time constraints and communication barriers were also identified in daily practice. These findings suggest that sustained efforts to promote collaboration, communication, awareness of the strengths and competencies of the various professional groups are essential for advancing interprofessional teamwork [68]. This is relevant as strengthening these key competencies underpins effective IPC, essential for both care quality and HCSPs’ work environment[69]. In the context of PHC, a clear understanding of roles and responsibilities within the healthcare team is pivotal to achieving optimal patient outcomes [10, 70]. Well-defined professional roles lead to better coordination, reduced duplication of efforts, and more effective utilisation of resources, ultimately enhancing the quality of care provided [71]. This role clarity is particularly important in PHC settings, where multidisciplinary teams are often required to address diverse health needs in a community-oriented manner [72]. Moreover, the ability to implement effective problem-solving strategies is crucial in PHC, particularly when addressing the complex and multifaceted health challenges faced by diverse populations. Collaborative decision-making, supported by evidence-based practices and tailored interventions, allows healthcare teams to navigate obstacles more efficiently, adapt to emerging health trends, and provide timely, patient-centred care [73, 74]. Moreover, teamwork in PHC is central to fostering a supportive and integrated care environment. A well-functioning team ensures that patients receive comprehensive care that addresses both medical and social determinants of health. An emphasis on role understanding, problem-solving approaches, and strong teamwork can improve the effectiveness of PHC delivery, contributing to better health outcomes and a more sustainable health system [73, 75].

A key focus of this study was on ensuring a psychological safe environment during simulation-based training [17, 76]. Such an environment allows learners to engage in realistic scenarios, make mistakes, and reflect without fear of judgement [76, 77]. To support this environment, and in line with standard practice at University of Applied Sciences Salzburg, tutors involved in the REALISE study were not the instructors responsible for grading students in other courses. This structural separation promotes via psychological safety open communication and controlled risk-taking [78], thereby fostering skill development and enhancing both technical and interprofessional abilities [77]. This approach boosts confidence and prepares HCSPs for complex clinical situations.

A further strength of the intervention was the close alignment of the case scenarios with real-world primary care practice, including their relevance in terms of disease prevalence. Effective implementation of such simulations, however, critically depends on trainers with expertise in simulation pedagogy, structured debriefing, and interprofessional facilitation, underscoring the importance of adequately qualified instructors for achieving meaningful educational outcomes [1, 15]. Therefore, one of the main strengths of this study lies in the implementation of high-quality simulation training, which, while resource-intensive, utilises evidence-based methods to ensure significant output. Moreover, the deliberate pairing of cases in a complementary fashion enabled participation from a wide range of professional groups within each learning path, reinforcing the interprofessional character of the training. Although the programme is resource-intensive –particularly regarding facilitators, simulation equipment, facilities, and participant time – findings indicate that long-term use in educational settings may be valuable in terms of outcomes and feasible for implementation [79]. Its modular design and blended-learning elements may help reduce in-person contact hours and associated resource demands [80]. Furthermore, embedding the programme into existing curricula and continuing professional education pathways may enhance efficiency and generate economies of scale [81]. In this context, the revised modules – featuring an expanded set of higher-quality e-learning components – will be incorporated into the curriculum of the Health Sciences programme at University of Applied Sciences Salzburg in the upcoming summer semester. Long-term economic benefits could arise from improved IPC, enhanced team performance, and potentially increased patient safety, which may indirectly reduce costs in routine healthcare delivery. Nevertheless, further research is required to conduct comprehensive cost–benefit analyses and to assess the sustainable implementation of the programme across diverse settings.

Future research should investigate how simulation-based interprofessional training can be tailored to accommodate diverse learning styles, prior experiences, and professional backgrounds of participants. In addition, studies should more explicitly examine how such training can reduce stereotypical perceptions between professional groups, and which pedagogical elements (e.g., role enactment, facilitated reflection, or mixed-experience teams) are most effective in achieving this, as stereotypes have been shown to significantly influence the effectiveness of IPE [53]. Further longitudinal research is also needed to assess the durability of competency gains, the transfer of interprofessional skills into routine primary care practice, and potential impacts on team performance, care processes, and patient-relevant outcomes. Such evidence is crucial for understanding the lasting impact of simulation-based training and for informing its sustainable integration into curricula and practice.

### Limitations

Several limitations of the study should be acknowledged. First, although the study was framed within PHC, GPs were not represented among the participants. Given the central role of GPs in coordinating primary care teams, their absence may limit the applicability of findings to fully representative PHC settings and the integration of interprofessional competencies into routine practice. In the current implementation of the modules at the University of Applied Sciences Salzburg, the role of GPs is addressed through collaborations with the local medical university and, where direct involvement is not feasible, substituted by appropriately trained standardized patients portraying physician to ensure high-fidelity simulation-based scenarios.

Second, the overall sample size was small (*n* = 18), with even smaller subgroups within some learning paths. This limits the statistical power to detect small or moderate effects and constrains the generalisability of the quantitative findings. While the study was designed as a feasibility investigation, caution is warranted when interpreting the results, particularly for subgroup comparisons and for extrapolation to larger populations of HCSPs.

Third, participant diversity presents a dual-edged issue. On one hand, the inclusion of professionals and students from multiple disciplines is a strength, reflecting the heterogeneity of real-world interprofessional teams. On the other hand, some professions were represented by only one or two participants, and not all predefined professional groups were included across all cohorts. This uneven representation may have limited the richness of interprofessional interactions for certain groups and reduces the ability to draw conclusions about the intervention’s effectiveness across all professional backgrounds.

Fourth, despite substantial recruitment efforts, enrolment in learning path 2 remained low, such that it could not be implemented. This might reflect several factors, including limited awareness and perceived relevance among students and HCSPs, scheduling conflicts with work or study commitments, the perceived time burden of participation, and limited institutional incentives. Without stronger cognitive anchoring and organizational support, well-designed educational offers may struggle to gain traction. Therefore, future strategies should prioritize early sensitization, integration into existing professional and academic structures, and flexible scheduling to enhance uptake.

Finally, as the intervention was conducted in a simulated setting rather than in participants’ routine work environments, the transferability of observed competency gains to everyday clinical practice remains to be established. Structural and organizational barriers in real-world primary care teams, such as time constraints, workflow limitations, and institutional policies, could influence the practical implementation of interprofessional skills.

Overall, these limitations highlight the need for future studies with larger, more balanced samples, inclusion of GPs, and assessment of real-world implementation to strengthen evidence for the scalability and generalisability of simulation-based interprofessional training in PHC.

## Conclusions

The findings indicate that the training intervention was feasible and well-received by participants from diverse professional backgrounds. The intervention effectively enhances practical skills such as team functioning and collaboration. These results showed that structured, simulation-based interprofessional training is a feasible and effective strategy for fostering interprofessional collaboration in PHC settings. Future programs should focus on integration of such training into routine practice, and long-term impact evaluation. In addition, future research should further explore the role of stereotypes in interprofessional learning settings.

## Supplementary Information

Supplementary Material 1. Table S1. Extension of the CONSORT statement for reporting on simulation research in healthcare. Abbreviations: ACLS: Advanced Cardiovascular Life Support, AHA: American Heart Association, BLS: Basic Life Support, COPD: Chronic Obstructive Pulmonary Disease, CRM: Crisis Resource Management, ECG: Electrocardiogram, HELLP: Hemolysis, Elevated Liver enzymes, Low Platelet count, QCPR: Quality Cardiopulmonary Resuscitation, SBAR: Situation, Background, Assessment, Recommendation, SOP: Standard Operating Procedure, TeamSTEPPS: Team Strategies and Tools to Enhance Performance and Patient Safety, VR: Virtual Reality.

Supplementary Material 2. Table S2. Allocation Scheme for Targeted Recruitment Based on Profession–Scenario Alignment.* Legend: ┼ Profession is relevant to the case scenario; ± Profession is potentially relevant to the case scenario; ─ Profession is not relevant to the case scenario.*

## Data Availability

The data that support the findings of this study are not openly available due to reasons of sensitivity but are available from the corresponding author upon reasonable request. Data are located under controlled access data storage at Salzburg University of Applied Sciences.

## Abbreviations

ART ANOVA: Aligned rank transform analysis of variance
HCSPs: Healthcare and social professionals
ICCAS: Interprofessional collaborative competency attainment survey
IPC: Interprofessional collaboration
IPE: : Interprofessional education
IPL: : Interprofessional learning
ISVS: Interprofessional socialization and valuing scale
PHC: Primary healthcare
S: Students
SUS: System usability scale
TAS: Teamwork assessment scale
